# Downregulated F-Box/LRR-Repeat Protein 7 Facilitates Pancreatic Cancer Metastasis by Regulating Snail1 for Proteasomal Degradation

**DOI:** 10.3389/fgene.2021.650090

**Published:** 2021-06-24

**Authors:** Liang Tang, Meng Ji, Xing Liang, Danlei Chen, Anan Liu, Guang Yang, Ligang Shi, Zhiping Fu, Chenghao Shao

**Affiliations:** Department of Pancreatic-Biliary Surgery, Changzheng Hospital, Second Military Medical University, Shanghai, China

**Keywords:** pancreatic cancer, FBXL7, BxPC-3 cell, EMT, Snail1

## Abstract

Pancreatic cancer (PCa) is one of the most aggressive lethal malignancies, and cancer metastasis is the major cause of PCa-associated death. F-box/LRR-repeat protein 7 (FBXL7) regulates cancer metastasis and the chemosensitivity of human pancreatic cancer. However, the clinical significance and biological role of FBXL7 in PCa have been rarely studied. In this study, we found that the expression of FBXL7 was down-regulated in PCa tissues compared with tumor-adjacent tissues, and the low expression of FBXL7 was positively associated with cancer metastasis. Functionally, overexpression of FBXL7 attenuated PANC1 cell invasion, whereas FBXL7 silencing promoted BxPC-3 cell invasion. Forced expression of FBXL7 upregulated the expression of epithelial markers (e.g., E-cadherin) and repressed the expression of mesenchymal markers (e.g., N-cadherin and Vimentin), indicating that FBXL7 negatively regulated the epithelial-mesenchymal transition (EMT) of PCa cells. Furthermore, we identified that FBXL7 repressed the expression of Snail1, a crucial transcription factor of EMT. Mechanistically, FBXL7 bound to Snail1 and promoted its ubiquitination and proteasomal degradation. *In vivo* studies demonstrated that FBXL7 inhibition promotes PCa metastasis. Taken together, our findings demonstrate that FBXL7 knockdown could efficiently enhance PCa metastasis by regulating Snail1-dependent EMT.

## Introduction

Pancreatic cancer (PCa) remains the fifth leading cause of cancer-related mortality in women and the fourth in men (Simoes et al., 2017), and has a dismal 5-year survival rate of less than 5% (Michl and Krug, 2018). Treatment for PCa includes chemotherapy, radiotherapy, surgery or the combined treatment (Li et al., 2004). Due to the concealed location, the early diagnosis of PCa is scarcely possible, and the PCa has metastasized when their diagnosed (Sun and Ma, 2019). Additionally, it isn’t easy to treat PCa due to the radio- and chemo-resistant nature of PCa. Therefore, exploring the underlying mechanisms of PCa progression is needed for novel therapies.

Protein degradation mediated by ubiquitin-proteasome is an important pathway regulating multiple biological processes, including post-transcriptional regulation, cell proliferation, tumorigenesis, and cancer metastasis (Baek, 2006). Protein ubiquitination is an orchestrated process, and its modification involves a series of reactions involving ubiquitin-activating enzymes (E1), ubiquitin-conjugating enzymes (E2), and ubiquitin ligases (E3) (Szczepanowski et al., 2005; Suresh et al., 2016). Mounting evidence demonstrated that the ubiquitination process is crucial for regulating oncogenesis and tumor progression of PCa (Jin et al., 2017; Ma et al., 2018). For example, F-box and WD repeat domain-containing 7 (FBW7) targets oncoprotein for ubiquitination and subsequent proteasomal degradation in PCa, resulting in the repression of PCa progression (Yada et al., 2004). Jin et al. (2017) reported that FBW7, as an E3 ligase, targets Enhancer of zeste homolog 2 (EZH2) for proteasomal degradation to inhibit PCa cell migration and invasion. Skp1-Cul1-F box (SCF) superfamily, acting as a member of E3 ligases, plays important role in different physiologic processes (Kitagawa and Kitagawa, 2016). Previous studies also revealed the regulatory role of F-box protein FBXW7 in PCa progression (Skaar et al., 2013). Ishii et al. (2017) identified the negative correlation of the decreased FBXW7 expression with poor prognosis in PCa. FBXW7 inhibition in PCa cells promotes cell proliferation, migration, and invasion by MCL1 accumulation (Ishii et al., 2017). F-box/LRR-repeat protein 7 (FBXL7) is also a member of the F-box protein family. Recent studies indicated that FBXL7 modulates chemotherapy-resistant in ovarian cancer and gastric cancer (Kamran et al., 2017). However, the function of FBXL7 in PCa remains unclear.

Metastasis is a major characteristic of malignancy tumors, and the epithelial-mesenchymal transition (EMT) is a key driver in promoting the migration and invasion of cancer cells (Neelakantan et al., 2017). As the core EMT regulatory factor, Twist1 and Snail1 are overexpressed in various cancers and induce EMT, which results in enhanced metastasis (Yoon et al., 2016). F-box proteins, such as FBXL14, target Snail for ubiquitination degradation and regulate EMT (Vinas-Castells et al., 2010). Here, we aimed to investigate the clinic pathologic significance of F-box proteins in PCa patients and found that FBXL7 suppressed PCa cell invasion *in vitro* and tumor metastasis *in vivo*. Furthermore, FBXL7 bound to Snail1 and promoted its ubiquitination and proteasomal degradation, and FBXL7 inhibition facilitating the PCa metastasis.

## Materials and Methods

### Human Tissue Specimens

The Institutional Ethics Committee of Changzheng hospital approved all sample collections (PCa tissues and tumor-adjacent tissues from PCa patients). There were 16 samples in each group. All patients underwent surgical treatment at Changzheng Hospital between 2018 and 2019. All patients provided informed consent before specimen collection. All patient characteristics were shown in Supplementary Table 1.

### Cell Culture

PCa cell lines CaPAN-1, BxPC-3, PANC-1, AsPC1, and normal human pancreatic ductal epithelial cells HPDE6-C7 were obtained from the ATCC (Manassas, VA, United States) and cultivated in Dulbecco’s modified eagle medium (DMEM) included with 10% heat-inactivated fetal bovine serum (FBS, GIBCO, Grand Island, NY, United States), 100 kU/L benzylpenicillin, and 100 mg/L streptomycin (Sigma-Aldrich, St. Louis, MO, United States). All cells were maintained in a 37°C incubator with 95% air and 5% CO_2_.

### Cell Transfection

#### FBXL7 Overexpression

Control plasmid pcDNA3.1 and recombinant plasmid pcDNA3.1-FBXL7 were purchased from Shanghai GenePharma Co., Ltd. (Shanghai, China) to overexpress FBXL7. A total of 10 μg of pcDNA3.1 or pcDNA3.1-FBXL7 was transfected into PANC1 cells by Lipofectamine 3000 (Invitrogen, Carlsbad, CA, United States) according to the instruction of this product specification.

#### ShRNAs

BxPC3 cells were transfected with FBXL7 shRNA or control-shRNA, synthesized by Genomeditech (Shanghai, China) to knockdown FBXL7. 5 μL titers of 1 × 10^8^ TU/mL FBXL7 shRNA or control-shRNA were added to each well (BxPC3 cells, 5 × 10^4^/ml). After transfection for 72 h, RT-PCR was carried out to assess the infection efficiency of FBXL7 knockdown. FBXL7 shRNA sequence were 5′-GCATCTCATCTGACGTGAGTT-3′.

### Reverse Transcription-Quantitative Polymerase Chain Reaction (RT-PCR)

Total RNAs of tissues (PCa and tumor-adjacent tissues) or cells (HPDE6-C7, CaPAN-1, BxPC-3, PANC-1, and AsPC1) were collected by TRIzol kit (Invitrogen) as per manufacturer′s standard, and then were reverse transcribed into cDNAs by PrimeScript RT reagent kit (Roche, Shanghai, China). Real-time PCR was performed using the SYBR Green Supermix (Invitrogen) on Applied Biosystems 7300 Real Time PCR system (Applied Biosystems, Foster City, CA) according to standard procedure. Thermocycling conditions were 95°C for 10 min, following by 35 cycles of 95°C for 10 s, 58°C for 15 s and 72°C for 20 s, and final 72°C for 20 min. Primers were listed as follows: FBXL7, 5′-CACGCAGCTCACCCACCTCTA-3′ (forward) and 5′-GGTGCAGTTCTTGGCGAGGT-3′ (reverse); Snail1, 5′-GAAAGGCCTTCAACTGCAAA; (forward) and 5′-TGACATCTGAGTGGGTCTGG (reverse); GAPDH, forward 5′-AGGTCGGAGTCAACGGATTTG-3′ (forward) and 5′-GTGAT GGCATGGACTGTGGTC-3′ (reverse). GAPDH expression served as an internal control, and the 2^–Δ^ ^Δ^ ^*Ct*^ method was applied to quantify FBXL7 expression.

### Immunohistochemical (IHC) Staining

IHC staining of PCa and matched tumor-adjacent tissues with antibodies against FBXL7 (ab59149, Abcam) were carried out according to the manufacturer’s declaration. In brief, the tissue specimens were fixed using formalin (10%) and embedded in paraffin. Serial sections of 5-μm thickness were sliced from the paraffin-embedded blocks. The slices were washed with TBS solution containing 0.025% Triton x-100 twice, and gently stir for 5 min each time. Then, TBS solution containing 10% normal serum and 1% BSA was used to seal at room temperature for 2 h. After incubating with FBXL7 antibody (8 μg/ml) overnight at 4°C, the sections were incubated with a Goat Anti-Rabbit IgG H&L (HRP) (1:5,000, ab205718, Abcam) at room temperature for 1 h. The sections used DAB-chromogen for development. Immunostained slides were photographed via a microscope and measured by Image J software.

### Western Blot

Cells (HPDE6-C7, CaPAN-1, BxPC-3, PANC-1, and AsPC1) were lysed via RIPA buffer (Beyotime, Shanghai, China) and protein was collected. Concentrations of total protein were quantified through a BCA Protein Assay Kit (Sigma-Aldrich). Equal amounts of each sample were separated via 12% sodium dodecyl sulfate-polyacrylamide gel electrophoresis (SDS-PAGE) and transmembrane to polyvinylidene fluoride (PVDF) membranes (Merck, Darmstadt, Germany). Immediately, the membranes were incubated with primary antibodies overnight at 4°C, which were FBXL7 (ab59149, Abcam; 1.25 μg/ml), E-Cadherin (ab231303, Abcam; 1 μg/ml), N-cadherin (ab18203, Abcam; 1 μg/ml), Vimentin (ab137321, Abcam; 1:3,000), Snail 1 (ab53519, Abcam; 3 μg/ml), Snail 2 (ab82846, Abcam; 1:500), ZEB 1 (ab155249, Abcam; 1:3,000), Twist (ab49254, Abcam; 2.5 μg/ml), and GAPDH (ab181602, Abcam; 1:10,000). Goat anti-Human IgG/HRP (Solarbio, SE101) was employed as secondary antibody to incubate the membranes. Finally, Positive signals were detected with an ECL luminescence reagent (Sangon Biotech, Shanghai, China).

### Cell Invasion and Migration Assays

Transwell assay was carried out for invasion assay (with Matrigel) and migration assay (without Matrigel) on 24-well culture plate (CLS3399-12EA, Corning Costar). BxPC-3, BxPC-3*^*FBXL*7^*^–^, PANC1 and PANC1*^*FBXL*7+^* cells (5 × 10^4^), which cultured with serum-free medium, were seeded into the upper chamber of 24-well plates. In contrast, the lower chamber was supplemented with complete medium containing 10% FBS. Cells, hatched at 37°C with 5% CO_2_ for 24 h, were stained with 0.1% crystal violet (Sigma-Aldrich) for 15 min. Cells were quantified with a microplate reader (Bio-Rad, Hercules, CA, United States).

### Co-immunoprecipitation (Co-IP) Assay

To obtain the BxPC3 cells, cells were scraped off 10 cm petri dishes and washed with cold PBS. The cells were lysed in RIPA buffer (Beyotime) contained with protease inhibitor cocktail (Solarbio, Beijing, China) at a cell density of 10^7^ cells/ml for half an hour. After centrifugation at 14,000 *g* for 15 min at 4°C, the supernatant was gathered. Protein concentrations were measured using the Bradford method and a standard protein curve was developed. Samples were pre-cleared through incubation with protein A + G agarose beads for half an hour on ice. Subsequently, 20 μg protein was incubated with rabbit antibody 5 μg IgG, anti-Snail antibody or anti-FBXL7 antibody. After washing with PBS thrice, compounds were added 60 μl 2 × loading buffer and boiled at 100°C for 5 min. Then, compounds and about 5 μg input were carried out by Western blot assays for protein expression.

### Lung Metastasis Model

All procedures involving experimental animals were approved by the Institutional Animal Ethics Committee of Changzheng hospital. Male nude mice (5-week old, 18–20 g, Total number: 20; *n* = 10 of each group) were purchased from Shanghai Model Organisms Center, Inc. (Shanghai, China) and kept in an animal room under SPF conditions, in a 12/12 h light/dark cycle, food and water were available *ad libitum*. FBXL7-knockdown BxPC-3 cells (shFBXL7/BxPC-3, 5 × 10^6^) stably expressing luciferase, resuspended in 100 μl of medium, were injected into tail vein of the mice. Mice were kept for 10 weeks and *in vivo* imaging system, a fluorescent detection system, were performed to capture the images for lung metastasis.

### Statistical Analysis

Statistical analyses were performed using SPSS 15.0 (SPSS, Version X; IBM, Armonk, NY, United States). The data are presented as the means ± standard deviation (SD). Comparisons between two groups were carried out using two-tailed Student’s *t*-test, while statistical differences among multiple groups were analyzed by ANOVA, followed by the Scheffé test. A *P*-value less than 0.05 was considered statistically significant.

## Results

### Clinic Pathologic Significance of FBXL7 in PCa Patients

**FIGURE 1:**
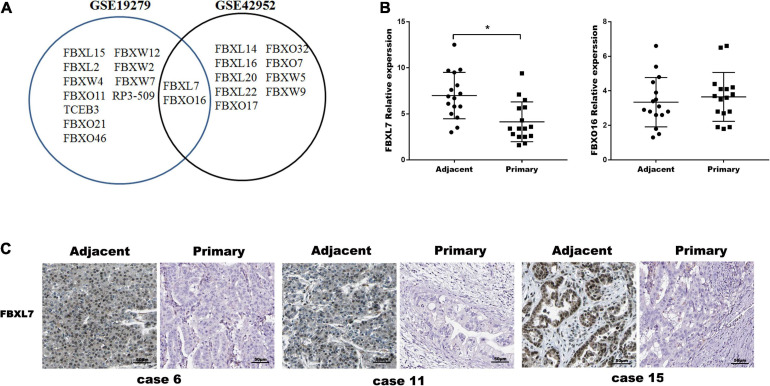
FBXL7 expressions Clinic pathologic significance of PCa patients. **(A)** Venn diagram showing the intersections of F-box genes between GSE19279 and GSE42952. **(B)** Relative expression of FBXL7 and FBXO16 in 16 pairs PCa tissues (*n* = 16) were detected by qRT-PCR. Data represent means ± SD of three independent experiments with significance determined by two-tailed Student’s *t*-test *P < 0.05. **(C)** Representative IHC staining showed positive expression of FBXL7 in adjacent tissues and negative expression of FBXL7 in tumor tissues (*n* = 5).

To investigate the putative role of F-box E3 ligase in PCa metastasis, human mRNA microarray GEO datasets (GSE19279 and GSE42952) were downloaded to analyze differential expressed mRNAs between normal pancreas and primary PCa tissues. Thirteen F-box mRNAs in GSE19279 and 11 F-box mRNAs in GSE42952 with significant changes (*P* < 0.001, Fold change ≥2 or ≤0.5) were filtered out. Venn diagram demonstrated the intersections of these genes between GSE19279 and GSE42952, and 2 co-differentially expressed F-box genes (up-regulated FBXO16 and down-regulated FBXL7) were found (Figure 1A).

The expression of FBXO16 and FBXL7 in PCa specimens was further assessed with qRT-PCR. The expression of FBXL7 in PCa specimens was lower than that in matched tumor-adjacent tissues (*n* = 16, *p* < 0.05). However, the expression of FBXO16 showed no significant difference in PCa tissues compared to tumor-adjacent tissues (Figure 1B). IHC staining was applied to assess the expression of FBXL7 in 16 pairs of PCa and tumor-adjacent tissues. 14 of 16 (87.5%) adjacent tissues showed a strongly positive expression of FBXL7, whereas FBXL7 signal was observed only 7 of 16 (44.0%) in PCa tissues (Figure 1C). These data suggested that FBXL7 may serve as a novel prognostic biomarker for PCa patients.

### FBXL7 Inhibition Enhanced Migration and Invasion of PCa Cells

**FIGURE 2:**
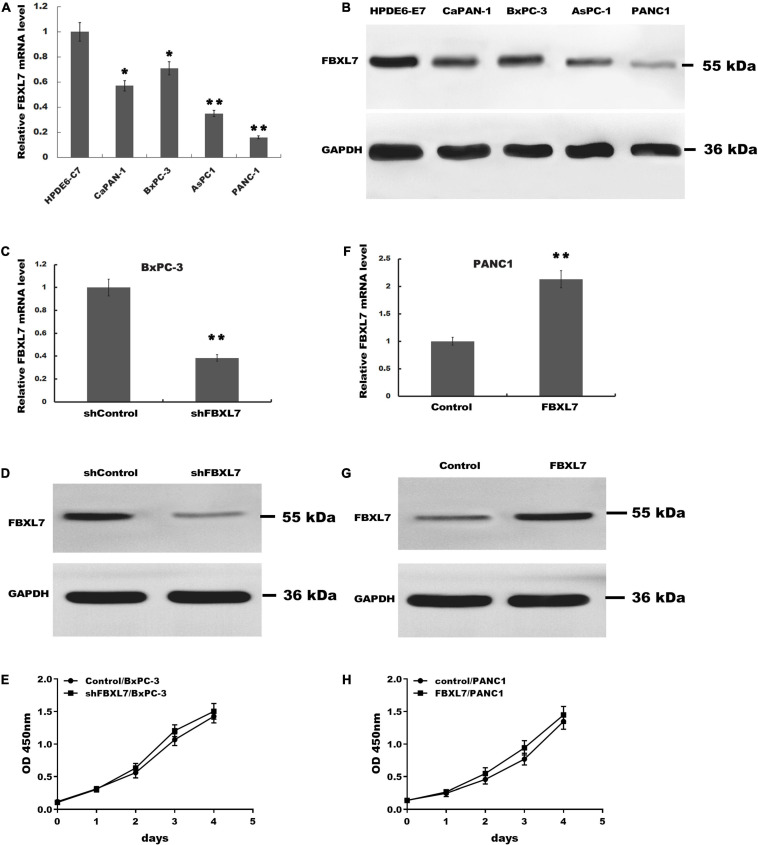
The proliferation of BxPC-3 and PANC-1 cells was not significantly altered after transfected with shFBXL7 or FBXL7. **(A,B)** The expression of FBXL7 mRNA in PCa cell lines (HPDE6-C7, CaPAN-1, BxPC-3, AsPC1, and PANC-1) were detected by RT-qPCR **(A)** and Western blot **(B)** analysis. **(C,D)** The expression of FBXL7 mRNA and protein were detected by RT-qPCR and Western blot analysis in stable FBXL7-silenced BxPC-3 cells. **(E)** The proliferation of FBXL7-silenced BxPC-3 cells was analyzed by CCK8 assay. **(F,G)** The expression of FBXL7 mRNA and protein were detected by RT-qPCR **(F)** and Western blot analysis **(G)** in FBXL7-overexpressing PANC1 cells. **(H)** The proliferation of stable FBXL7-overexpressing PANC1 cells was analyzed by CCK8 assay. The figure **(A,C,F)** Data represent means ± SD of three independent experiments with significance determined by two-tailed Student’s *t*-test *P < 0.05, **P < 0.01.

**FIGURE 3:**
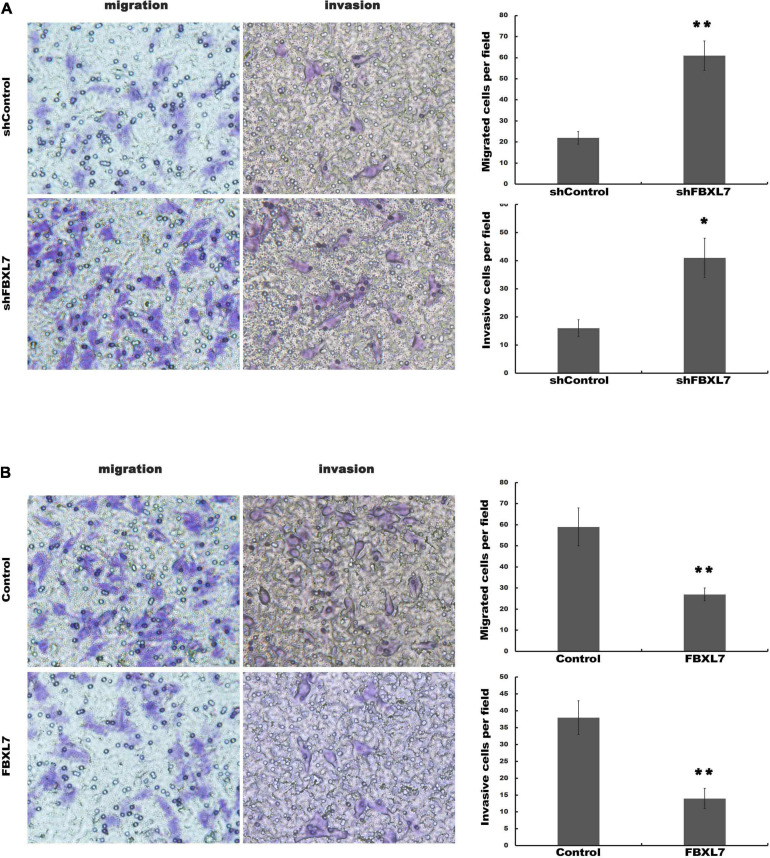
FBXL7 inhibition enhanced migration and invasion of PCa cells. **(A)** The BxPC-3 cell migration and invasion were detected after shFBXL7 transfection by Transwell assay. **(B)** The PANC1 cell migration and invasion were detected after FBXL7 overexpression by Transwell assay. Data represent means ± SD of three independent experiments with significance determined by two-tailed Student’s *t*-test *P < 0.05, **P < 0.01.

Next, the expression of FBXL7 was assessed in normal human pancreatic ductal epithelial cells (HPDE6-C7) and four PCa cell lines (CaPAN-1, BxPC-3, AsPC1, and PANC1 cells). Compared with HPDE6-C7 cells, the expression of FBXL7 in four PCa cell lines was significantly increased. The expression level of FBXL7 was highest in BXPC-3 cells and lower in PANC1 cells (Figures 2A,B). Therefore, FBXL7 was knock-downed in BxPC-3 cells and overexpressed in PANC1 cells to investigate its function. The efficiency of FBXL7 knockdown and overexpression was confirmed by RT-PCR and western blot (Figures 2C,D,F,G). The proliferation of cells was detected by CCK8 assay. As shown in Figures 2E,H, the proliferation of FBXL7 knockdown and overexpression cells over 96 h was not significantly different compared to the control cells. However, FBXL7 knockdown in BxPC-3 cells significantly promoted cell migration and invasion compared with control (Figure 3A). In accord with the loss-of-function studies in BxPC-3 cells, gain-of-function studies by overexpression of FBXL7 in PANC1 showed a significantly decreased cell migration and invasion (Figure 3B). These experimental results suggested that FBXL7 acts as a tumor suppressor in PCa progression.

### FBXL7 Repressed the Epithelial-Mesenchymal Transition (EMT) Process of PCa

**FIGURE 4:**
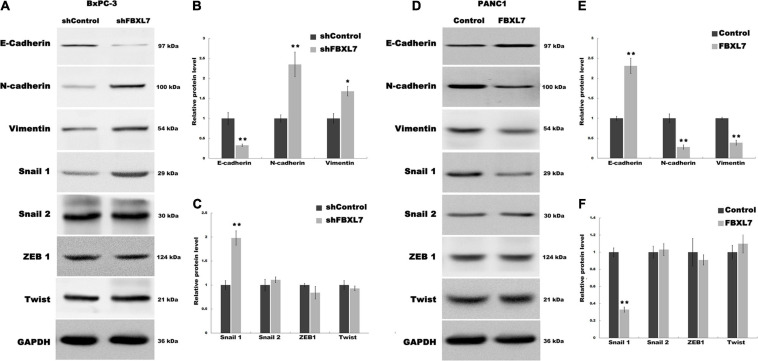
FBXL7 regulates the EMT process of PCa. **(A–C)** The expression of E-cadherin, N-cadherin, Vimentin, Snail1, Snail2, ZEB1 and Twist protein was detected by Western blot analysis in BxPC-3 cells after shFBXL7 transfection. **(D–F)** The expression of E-cadherin, N-cadherin, Vimentin, Snail1, Snail2, ZEB1 and Twist protein was detected by Western blot analysis in PANC1 cells after FBXL7 overexpression. Data represent means ± SD of three independent experiments with significance determined by two-tailed Student’s *t*-test *P < 0.05, **P < 0.01.

EMT is associated with cell invasion and cancer metastasis (Diepenbruck and Christofori, 2016). To confirm whether FBXL7 suppresses the EMT of PCa cells to prevent cell migration and invasion, we explored the regulatory role of FBXL7 in the expression of epithelial markers (e.g., E-cadherin) and mesenchymal markers (e.g., N-cadherin and Vimentin). As shown in Figures 4A–C, FBXL7 knockdown in BxPC-3 cells significantly decreased the E-cadherin expression level and increased the Vimentin and N-cadherin expression levels. Alternatively, FBXL7 overexpression in PANC1 cells up-regulated the expression of E-cadherin and down-regulated the expression of Vimentin and N-cadherin (Figures 4D–F), indicating that FBXL7 negatively regulated the EMT of PCa cells.

**FIGURE 5:**
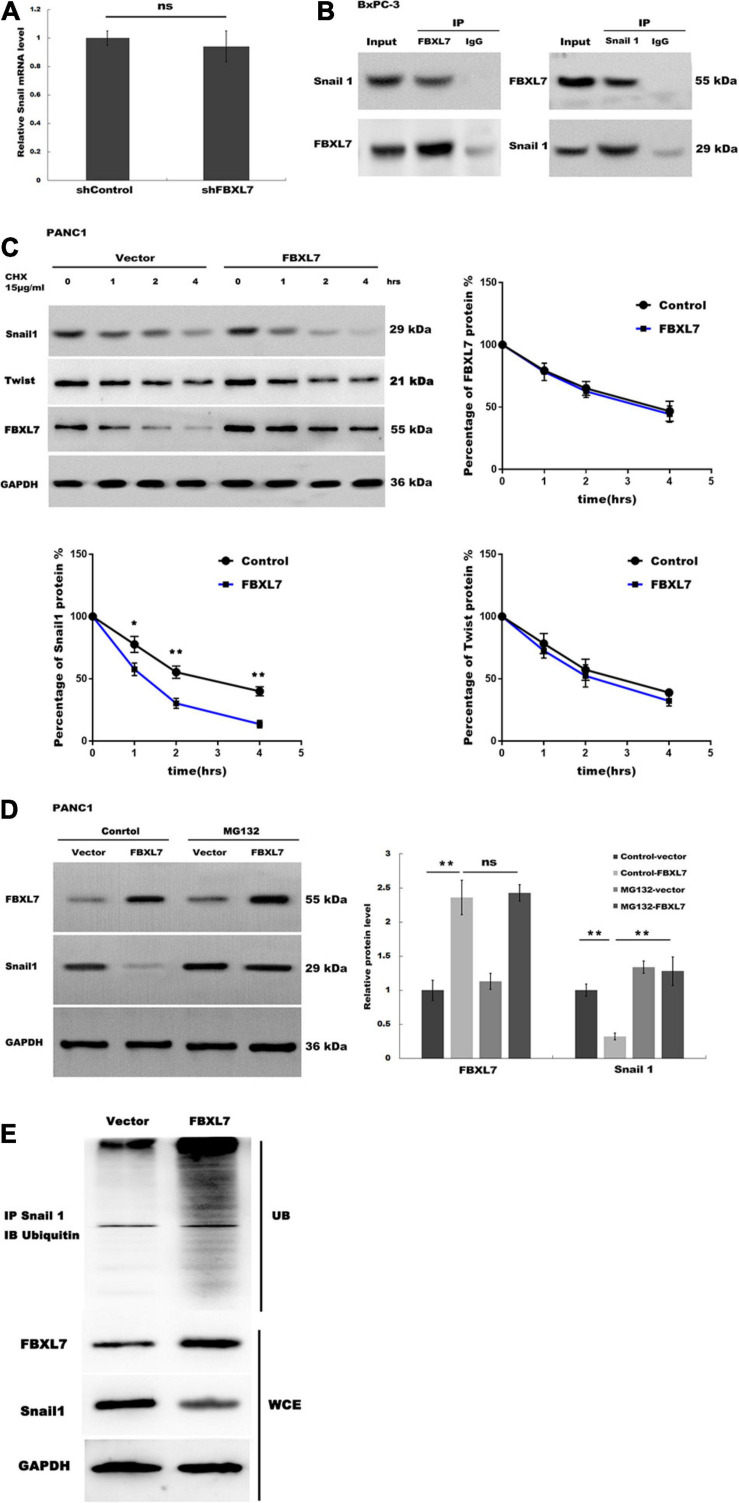
FBXL7 induces Snail 1 ubiquitination and proteasomal degradation. **(A)** The expression of Snail1 mRNA was detected in BxPC-3 cells with or without FBXL7 knockdown by RT-qPCR analysis. **(B)** The relationship between FBXL7 and Snail1 was detected by co-IP assay using anti-FBXL7 or anti-Snail1 antibody in BxPC-3 cells. **(C)** The expression of Snail1, Twist,and FBXL7 was detected in PANC1 cells overexpressed with FBXL7 at 0, 1, 2, and 4 h by cycloheximide chase assay. Data represent means ± SD of three independent experiments with significance determined by two-tailed Student’s *t*-test *P < 0.05, **P < 0.01. **(D)** The expression of SNAIL1 was detected in PANC1 cells transduced with or without FBXL and applied MG132 by Western blot. **(E)** FBXL7 increases the ubiquitination of Snail1. Control and FBXL7 infected cell lysates were immunoprecipitated with anti-Snail1 antibody. The IP was then analyzed by immunoblotting with anti-ubiquitin antibody. WCE = whole cell extract. Data represent means ± SD of three independent experiments with significance determined by one-way ANOVA followed by the Scheffé test. **P < 0.01.

To further expound the mechanism that FBXL7 regulates the EMT of PCa cells, we explored 4 upstream transcription factors, Snail 1, Snail 2 (Wawruszak et al., 2019), ZEB1 (Goossens et al., 2017; Cho et al., 2019), and Twist (Rout-Pitt et al., 2018), which have an essential role in regulating EMT. Figure 5A showed that the expression of Snail 1 was obviously up-regulated following FBXL7 knockdown in BxPC-3 cells. In accord with the loss-of-function studies in BxPC-3 cells, gain-of-function studies by overexpression of FBXL7 in PANC1 showed that the Snail 1 expression level was decreased following FBXL7 overexpression (Figure 5B). There was no significant change in the expression of Snail 2, ZEB1, and Twist after FBXL7 knockdown or overexpression (Figures 4A,C,D,F). Thus, we deduce that FBXL7 suppresses the EMT of PCa cells by down-regulating Snail 1 expression.

### FBXL7 Induced Snail1 Ubiquitination and Proteasomal Degradation

Recent studies have demonstrated that the E3 ligase FBXL7 induces ubiquitination of target protein, leading to proteasome-dependent degradation (Coon et al., 2012; Liu et al., 2015). Our data showed that FBXL7 knockdown did not affect Snail1 mRNA levels (Figure 5A). We speculated that FBXL7 might bind to Snail1, leading to its ubiquitination and proteasomal degradation. Therefore, we detected the interaction between FBXL7 and Snail1 in BxPC-3 cells by reciprocal Co-IP. As shown in Figure 5B, a positive Snail1 signal was observed in the Co-IP pulled down by an anti-FBXL7-specific antibody. Meanwhile, FBXL7 was also detected in the protein pool pulled down by the anti-Snail1 antibody. These results confirmed there is physical interaction between FBXL7 and Snail1 in BxPC-3 cells.

Next, we assessed the expression of Snail1 and Twist in PANC1 cells overexpressed with FBXL7 at 0, 1, 2, and 4 h by cycloheximide (CHX) chase assay. Figure 5C showed that the half-life of Snail1 was obviously decreased in the FBXL7-overexpressing PANC1 cells compared to the control cells, but the half-life of Twist was no significant change in the FBXL7-overexpressing PANC1 cells compared to the control cells, Furthermore, we applied a proteasome specific inhibitor (MG132) to treat FBXL7-overexpressing or control PANC1 cells. Figure 5D showed that the decreased expression of Snail1 was blocked after MG132 treatment. suggesting that the ubiquitin-proteasome system was involved in Snail1 degradation. To validate this, we detected polyubiquitination of Snail1 with or without FBXL7 expression. Indeed, knockdown of FBXL7decreased polyubiquitination of Snail1 (Figure 5E). Collectively, these data suggested that FBXL7 binds to Snail1 and induces the down-regulation of Snail1 by promoting its ubiquitination and proteasomal degradation.

### FBXL7 Inhibition Facilitated Tumor Metastasis *in vivo*

**FIGURE 6:**
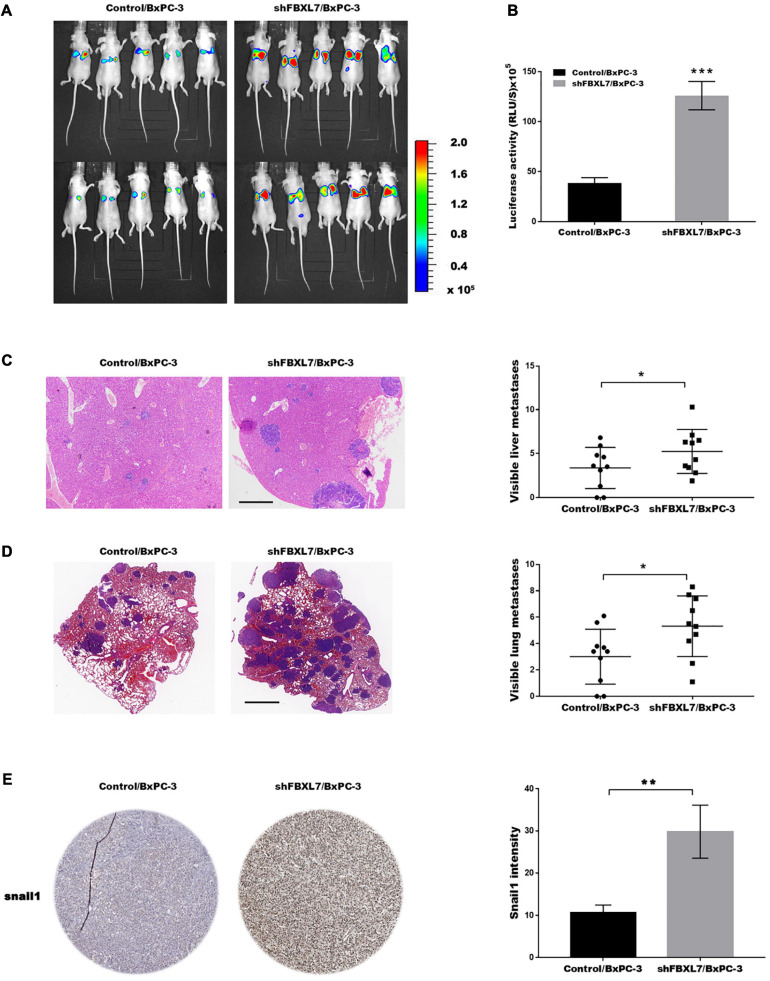
FBXL7 inhibition facilitated tumor metastasis *in vivo.* **(A,B)** Cancer metastasis was assayed using bioluminescent imaging after FBXL knockdown. **(C)** After repressing FBXL7, the incidence and number of visible metastases in liver was assayed. Scale bar = 1 mm (*n* = 10). **(D)** After repressing FBXL7, the incidence and number of visible metastases per lung in each cohort following inoculation was assayed. Scale bar = 1 mm (*n* = 10). **(E)** IHC staining for snail1. Data represent means ± SD The significance determined by two-tailed Student’s *t*-test. *P < 0.05, ***P < 0.01.

Based on the above findings that FBXL7 suppressed PCa cell migration and invasion, we explored the effects of FBXL7 on cancer metastatic ability *in vivo*. FBXL7-knockdown BxPC-3 cells (shFBXL7/BxPC-3) stably expressing luciferase were injected into the mice’s tail vein. The bioluminescent signal was detected at 10 weeks after tail vein injection. Figures 6A,B showed that the incidence of lung metastasis in the shFBXL7/BxPC-3 group is obviously increased, compared with the control group. The metastasis of FBXL7 was also confirmed in mice lung and liver by H&E staining, as shown in Figures 6C,D; the FBXL7 knockdown obviously increased the liver and lung metastasis of BxPC-3 cell compared with the control group. In addition, the expression of Snail 1 was significantly increased in the FBXL7 knock-down group (Figure 6E). These data suggested that the inhibition FBXL7 promotes pancreatic cancer cell invasion *in vitro* and tumor metastasis *in vivo.*

## Discussion

In the present study, we investigated the role of FBXL7 in regulating PCa cell invasion *in vitro* and PCa metastasis *in vivo*. The available data demonstrate that (i) The FBXL7 expression is decreased in PCa tissues compared to adjacent controls, (ii) FBXL7 inhibition enhanced migration and invasion of PCa cells, (iii) FBXL7 represses the EMT process of PCa, (iv) FBXL7 induces Snail 1 ubiquitination and proteasomal degradation, and (v) FBXL7 inhibition facilitated tumor metastasis *in vivo*. These results verified the important role of FBXL7 in regulating PCa metastasis, identified its underlying mechanism, and may provide a new opportunity for PCa therapy.

To maintain multiple basic biological processes, ubiquitin-dependent proteolysis is commonly necessary for the recycling of amino acids and the elimination of misfolded or damaged proteins (Song et al., 2019). F-box proteins are key components of the SCF (SKP1-CUL1-F-box) E3 ubiquitin ligases and participate in the ubiquitin-dependent protein degradation (Wang et al., 2017). Mounting evidences have demonstrated that aberrant F-box protein or F-box protein-mediated protein degradation is closely correlated with tumorigenesis and cancer progression of certain types of human cancers (Sun et al., 2018; Galindo-Moreno et al., 2019). For example, Li et al. (2016) showed that FBXW7 promotes gastric cancer cell apoptosis and inhibits EMT via targeting Snail1 and ZEB1 for proteasomal degradation. FBXO11 promotes breast cancer progression via ubiquitination and degradation of Snail family proteins (Jin et al., 2015). FBXL5 and FBXL14 also act as tumor suppressor genes in gastric cancer, cervical cancer, and head and neck cancer through degrading Snail1 and inhibiting EMT (Wu et al., 2015; Yang et al., 2016).

FBXL7 is a newly identified SCF E3 ubiquitin ligase. Coon et al. (2012) reported for the first time the biological function of FBXL7 in regulating cell proliferation. They demonstrated that FBXL7 represses epithelial cell proliferation by degrading Aurora A. Liu et al. (2015) showed that FBXL7 regulates mitochondrial function via targeting survivin for proteasomal degradation. Kamran et al. (2017) further demonstrated that Aurora A inhibits FBXL7 expression, and thus destroys the role of FBXL7 in mediating ubiquitination and proteasomal degradation of survival in gastric cancer. Their study suggested that FBXL7 acts as a tumor suppressor gene in gastric cancer. On the contrary, another study reported that FBXL7 overexpression in ovarian cancer is correlated with chemoresistance and predicts a poor prognosis of ovarian cancer (Chiu et al., 2018). The biological role of FBXL7 is still controversial in different types of cancers and is unknown in PCa.

To reveal the effect of FBXL7 on PCa progression, we first assessed the expression of FBXL7 in PCa. Consistent with previous results (GSE19279 and GSE42952), the expression level of FBXL7 is significantly downregulated in PCa tissues compared with adjacent normal tissues. Gain- and loss-of-function studies demonstrated that FBXL7 overexpression results in significantly decreased cell migration and invasion, whereas FBXL7 knockdown promotes cell migration and invasion compared with control. Mounting evidences have revealed the role of F-box proteins in regulating EMT in multiple types of tumors, including FBXW7 in lung cancer and gastric cancer (Li et al., 2016; Zhang et al., 2018), FBXO11 in breast cancer and gastric cancer (Zheng et al., 2014; Jin et al., 2015), and FBXO45 in prostate cancer and PCa (Abshire et al., 2016; Wang et al., 2018). We thus investigated whether FBXL7 regulates PCa EMT. FBXL7 knockdown inhibits E-cadherin expression, and increases Vimentin and N-cadherin expression, whereas FBXL7 overexpression increases E-cadherin expression and decreases Vimentin and N-cadherin expression, indicating that FBXL7 represses the EMT of PCa cells. Furthermore, to clarify the mechanism that FBXL7 regulates the EMT of PCa cells, we assessed the regulatory role of FBXL7 in 4 upstream transcription factors, Snail 1, Snail 2, ZEB1, and Twist. The current data showed that FBXL7 binds to Snail1 and induces the down-regulation of Snail1 by promoting its ubiquitination and proteasomal degradation. Interestingly, another study reported that FBXL7 knockdown in two different cells lines (SW620 and MCF-7) does not significantly increased the expression of Snail1 (Vinas-Castells et al., 2014). In their study, FBXL7 knockdown slightly increased the expression of Snail1 in SW620 cells, but had no effect at all in MCF-7 cells. In our study, FBXL7can significantly increase the expression of Snail1in BxPC-3 cells. Therefore, we believe that FBXL7 has different regulatory functions on Snail1 in different tumor cells. Taken together, our results demonstrated that FBXL7 represses EMT and metastasis of PCa by targeting Snail1 for proteasomal degradation.

## Data Availability Statement

The original contributions presented in the study are included in the article/Supplementary Material, further inquiries can be directed to the corresponding author/s.

## Ethics Statement

The studies involving human participants were reviewed and approved by the Institutional Ethics Committee of Changzheng Hospital. The patients/participants provided their written informed consent to participate in this study. The animal study was reviewed and approved by the Institutional Ethics Committee of Changzheng Hospital.

## Author Contributions

LT, MJ, and XL performed the experiments, analyzed the data, and wrote the manuscript. DC and AL conceived the study. GY and LS initiated the study. ZF was responsible for repairing this manuscript. CS finally agreed to the manuscript. All authors reviewed the manuscript.

## Conflict of Interest

The authors declare that the research was conducted in the absence of any commercial or financial relationships that could be construed as a potential conflict of interest.

## References

[B1] Abshire C. F. Carroll J. L. Dragoi A. M. (2016). FLASH protects ZEB1 from degradation and supports cancer cells’ epithelial-to-mesenchymal transition. *Oncogenesis* 5:e254. 10.1038/oncsis.2016.55 27526108 PMC5007829

[B2] Baek K. H. (2006). Cytokine-regulated protein degradation by the ubiquitination system. *Curr. Protein Pept. Sci.* 7 171–177. 10.2174/138920306776359740 16611142

[B3] Chiu H. W. Chang J. S. Lin H. Y. Lee H. H. Kuei C. H. Lin C. H. (2018). FBXL7 Upregulation predicts a poor prognosis and associates with a possible mechanism for paclitaxel resistance in ovarian cancer. *J. Clin. Med.* 7:330. 10.3390/jcm7100330 30301218 PMC6209951

[B4] Cho E. S. Kang H. E. Kim N. H. Yook J. I. (2019). Therapeutic implications of cancer epithelial-mesenchymal transition (EMT). *Arch. Pharm. Res.* 42 14–24. 10.1007/s12272-018-01108-7 30649699

[B5] Coon T. A. Glasser J. R. Mallampalli R. K. Chen B. B. (2012). Novel E3 ligase component FBXL7 ubiquitinates and degrades Aurora A, causing mitotic arrest. *Cell Cycle* 11 721–729. 10.4161/cc.11.4.19171 22306998 PMC3318106

[B6] Diepenbruck M. Christofori G. (2016). Epithelial-mesenchymal transition (EMT) and metastasis: yes, no, maybe? *Curr. Opin. Cell. Biol.* 43 7–13. 10.1016/j.ceb.2016.06.002 27371787

[B7] Galindo-Moreno M. Giraldez S. Limon-Mortes M. C. Belmonte-Fernandez A. Reed S. I. Saez C. (2019). SCF(FBXW7)-mediated degradation of p53 promotes cell recovery after UV-induced DNA damage. *FASEB J.* 33 11420–11430. 10.1096/fj.201900885r 31337255 PMC6766643

[B8] Goossens S. Vandamme N. Van Vlierberghe P. Berx G. (2017). EMT transcription factors in cancer development re-evaluated: beyond EMT and MET. *Biochim. Biophys. Acta Rev. Cancer* 1868 584–591. 10.1016/j.bbcan.2017.06.006 28669750

[B9] Ishii N. Araki K. Yokobori T. Gantumur D. Yamanaka T. Altan B. (2017). Reduced FBXW7 expression in pancreatic cancer correlates with poor prognosis and chemotherapeutic resistance via accumulation of MCL1. *Oncotarget* 8 112636–112646. 10.18632/oncotarget.22634 29348852 PMC5762537

[B10] Jin X. Yang C. Fan P. Xiao J. Zhang W. Zhan S. (2017). CDK5/FBW7-dependent ubiquitination and degradation of EZH2 inhibits pancreatic cancer cell migration and invasion. *J. Biol. Chem.* 292 6269–6280. 10.1074/jbc.m116.764407 28242758 PMC5391756

[B11] Jin Y. Shenoy A. K. Doernberg S. Chen H. Luo H. Shen H. (2015). FBXO11 promotes ubiquitination of the Snail family of transcription factors in cancer progression and epidermal development. *Cancer Lett.* 362 70–82. 10.1016/j.canlet.2015.03.037 25827072 PMC4406488

[B12] Kamran M. Long Z. J. Xu D. Lv S. S. Liu B. Wang C. L. (2017). Aurora kinase A regulates Survivin stability through targeting FBXL7 in gastric cancer drug resistance and prognosis. *Oncogenesis* 6:e298. 10.1038/oncsis.2016.80 28218735 PMC5337621

[B13] Kitagawa K. Kitagawa M. (2016). The SCF-type E3 Ubiquitin ligases as cancer targets. *Curr. Cancer Drug. Targets* 16 119–129. 10.2174/1568009616666151112122231 26560120

[B14] Li D. Xie K. Wolff R. Abbruzzese J. L. (2004). Pancreatic cancer. *Lancet* 363 1049–1057. 15051286 10.1016/S0140-6736(04)15841-8

[B15] Li H. Wang Z. Zhang W. Qian K. Xu W. Zhang S. (2016). Fbxw7 regulates tumor apoptosis, growth arrest and the epithelial-to-mesenchymal transition in part through the RhoA signaling pathway in gastric cancer. *Cancer Lett.* 370 39–55. 10.1016/j.canlet.2015.10.006 26458995

[B16] Liu Y. Lear T. Iannone O. Shiva S. Corey C. Rajbhandari S. (2015). The proapoptotic F-box protein Fbxl7 regulates mitochondrial function by mediating the ubiquitylation and proteasomal degradation of survivin. *J. Biol. Chem.* 290 11843–11852. 10.1074/jbc.m114.629931 25778398 PMC4424325

[B17] Ma T. Chen W. Zhi X. Liu H. Zhou Y. Chen B. W. (2018). USP9X inhibition improves gemcitabine sensitivity in pancreatic cancer by inhibiting autophagy. *Cancer Lett.* 436 129–138. 10.1016/j.canlet.2018.08.010 30118840

[B18] Michl P. Krug S. (2018). Overcoming immune evasion in pancreatic cancer: the combination matters. *Gut* 67 997–999. 10.1136/gutjnl-2017-315443 29217750

[B19] Neelakantan D. Zhou H. Oliphant M. U. J. Zhang X. Simon L. M. Henke D. M. (2017). EMT cells increase breast cancer metastasis via paracrine GLI activation in neighbouring tumour cells. *Nat. Commun.* 8:15773. 10.1038/ncomms15773 PMC5472791 28604738

[B20] Rout-Pitt N. Farrow N. Parsons D. Donnelley M. (2018). Epithelial mesenchymal transition (EMT): a universal process in lung diseases with implications for cystic fibrosis pathophysiology. *Respir. Res.* 19:136. 10.1186/s12931-018-0834-8 PMC6052671 30021582

[B21] Simoes P. K. Olson S. H. Saldia A. Kurtz R. C. (2017). Epidemiology of pancreatic adenocarcinoma. *Chin. Clin. Oncol.* 6:24. 10.21037/cco.2017.06.32 28705001

[B22] Skaar J. R. Pagan J. K. Pagano M. (2013). Mechanisms and function of substrate recruitment by F-box proteins. *Nat. Rev. Mol. Cell Biol.* 14 369–381. 10.1038/nrm3582 23657496 PMC3827686

[B23] Song Y. Lin M. Liu Y. Wang Z. W. Zhu X. (2019). Emerging role of F-box proteins in the regulation of epithelial-mesenchymal transition and stem cells in human cancers. *Stem Cell Res. Ther.* 10:124. 10.1186/s13287-019-1222-0 PMC6472071 30999935

[B24] Sun X. Wang T. Guan Z. R. Zhang C. Chen Y. Jin J. (2018). FBXO2, a novel marker for metastasis in human gastric cancer. *Biochem. Biophys. Res. Commun.* 495 2158–2164. 10.1016/j.bbrc.2017.12.097 29269301

[B25] Sun Y. Ma L. (2019). New insights into long non-coding RNA MALAT1 in cancer and metastasis. *Cancers (Basel)* 11:216. 10.3390/cancers11020216 30781877 PMC6406606

[B26] Suresh B. Lee J. Kim K. S. Ramakrishna S. (2016). The importance of ubiquitination and deubiquitination in cellular reprogramming. *Stem Cells Int.* 2016:6705927. 10.1155/2016/6705927 PMC4736574 26880980

[B27] Szczepanowski R. H. Filipek R. Bochtler M. (2005). Crystal structure of a fragment of mouse ubiquitin-activating enzyme. *J. Biol. Chem.* 280 22006–22011. 10.1074/jbc.m502583200 15774460

[B28] Vinas-Castells R. Beltran M. Valls G. Gomez I. Garcia J. M. Montserrat-Sentis B. (2010). The hypoxia-controlled FBXL14 ubiquitin ligase targets SNAIL1 for proteasome degradation. *J. Biol. Chem.* 285 3794–3805. 10.1074/jbc.m109.065995 19955572 PMC2823521

[B29] Vinas-Castells R. Frias A. Robles-Lanuza E. Zhang K. Longmore G. D. Garcia de Herreros A. (2014). Nuclear ubiquitination by FBXL5 modulates Snail1 DNA binding and stability. *Nucleic Acids Res.* 42 1079–1094. 10.1093/nar/gkt935 24157836 PMC3902928

[B30] Wang F. F. Zhang X. J. Yan Y. R. Zhu X. H. Yu J. Ding Y. (2017). FBX8 is a metastasis suppressor downstream of miR-223 and targeting mTOR for degradation in colorectal carcinoma. *Cancer Lett.* 388 85–95. 10.1016/j.canlet.2016.11.031 27916606

[B31] Wang K. Qu X. Liu S. Yang X. Bie F. Wang Y. (2018). Identification of aberrantly expressed F-box proteins in squamous-cell lung carcinoma. *J. Cancer Res. Clin. Oncol.* 144 1509–1521. 10.1007/s00432-018-2653-1 29728763 PMC11813518

[B32] Wawruszak A. Kalafut J. Okon E. Czapinski J. Halasa M. Przybyszewska A. (2019). Histone deacetylase inhibitors and phenotypical transformation of cancer cells. *Cancers (Basel)* 11:148. 10.3390/cancers11020148 30691229 PMC6406474

[B33] Wu W. Ding H. Cao J. Zhang W. (2015). FBXL5 inhibits metastasis of gastric cancer through suppressing Snail1. *Cell Physiol. Biochem.* 35 1764–1772. 10.1159/000373988 25832584

[B34] Yada M. Hatakeyama S. Kamura T. Nishiyama M. Tsunematsu R. Imaki H. (2004). Phosphorylation-dependent degradation of c-Myc is mediated by the F-box protein Fbw7. *EMBO J.* 23 2116–2125. 10.1038/sj.emboj.7600217 15103331 PMC424394

[B35] Yang W. H. Su Y. H. Hsu W. H. Wang C. C. Arbiser J. L. Yang M. H. (2016). Imipramine blue halts head and neck cancer invasion through promoting F-box and leucine-rich repeat protein 14-mediated Twist1 degradation. *Oncogene* 35 2287–2298. 10.1038/onc.2015.291 26257063 PMC5929114

[B36] Yoon N. A. Jo H. G. Lee U. H. Park J. H. Yoon J. E. Ryu J. (2016). Tristetraprolin suppresses the EMT through the down-regulation of Twist1 and Snail1 in cancer cells. *Oncotarget* 7 8931–8943. 10.18632/oncotarget.7094 26840564 PMC4891015

[B37] Zhang Y. Zhang X. Ye M. Jing P. Xiong J. Han Z. (2018). FBW7 loss promotes epithelial-to-mesenchymal transition in non-small cell lung cancer through the stabilization of Snail protein. *Cancer Lett.* 419 75–83. 10.1016/j.canlet.2018.01.047 29355657

[B38] Zheng H. Shen M. Zha Y. L. Li W. Wei Y. Blanco M. A. (2014). PKD1 phosphorylation-dependent degradation of SNAIL by SCF-FBXO11 regulates epithelial-mesenchymal transition and metastasis. *Cancer Cell* 26 358–373. 10.1016/j.ccr.2014.07.022 25203322 PMC4159622

