# Supplementary material for: Downregulated F-Box/LRR-Repeat Protein 7 Facilitates Pancreatic Cancer Metastasis by Regulating Snail1 for Proteasomal Degradation
Source: Front Genet. 2021 Jun 24;12:650090. doi: 10.3389/fgene.2021.650090 (PMC8264591; doi:10.3389/fgene.2021.650090)
Supplement: Supplementary file 1 [file Table_1.docx]

**Supplement Table 1. Correlation between FBXL7 level and clinicopathological characteristics of PCa**

| **Characteristics** | **n** | **FBXL7 expression**  **Low High** | | ***p value*** | |
| --- | --- | --- | --- | --- | --- |
| **Total cases**  **Gender**  Male  Female  **Age**  ≤55  >55  **TNM stage**  I+II  III+IV | 16  10  6  7  9  6  10 | 8  6  3  3  5  2  4 | 8  4  3  4  4  4  6 | 0.562  0.716  0.437 |  |
| **Tumor size**  ≤3  >3 | 7  9 | 3  4 | 4  5 | 0.832 |  |
