# Supplementary material for: Downregulated F-Box/LRR-Repeat Protein 7 Facilitates Pancreatic Cancer Metastasis by Regulating Snail1 for Proteasomal Degradation
Source: Front Genet. 2021 Jun 24;12:650090. doi: 10.3389/fgene.2021.650090 (PMC8264591; doi:10.3389/fgene.2021.650090)

**Supplementary data**

1. FBXL7 expression didn’t correlatives with reduced survival of the PAAD patients


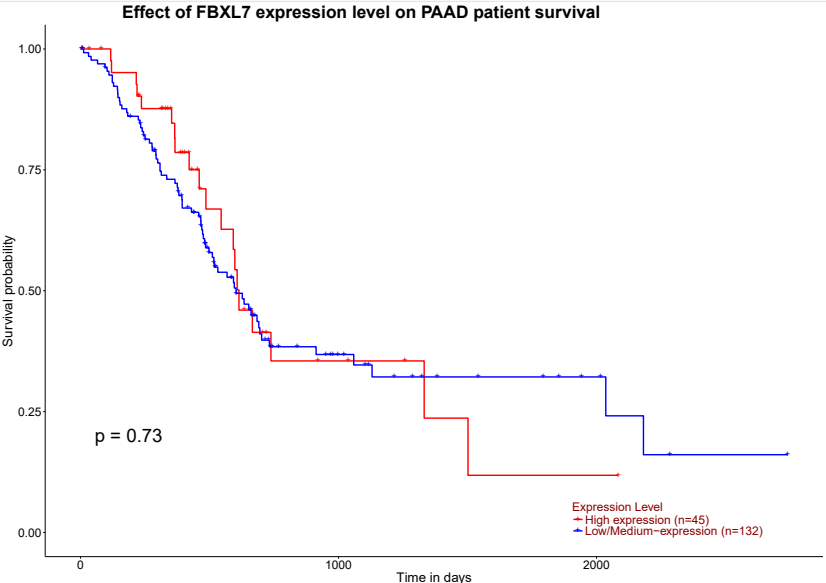


1. The lower magnification of FBXL7 in case 6, 11 and 15


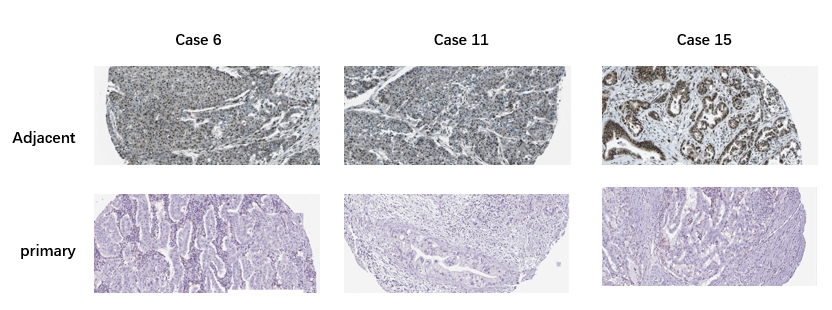


1. FBXL7 didn’t regulate the phosphorylation of Snail1


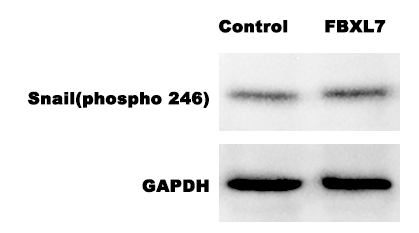


1. The expression of FBXL5, a major Snail1 regulators, in Pancreatic cancer cell lines.


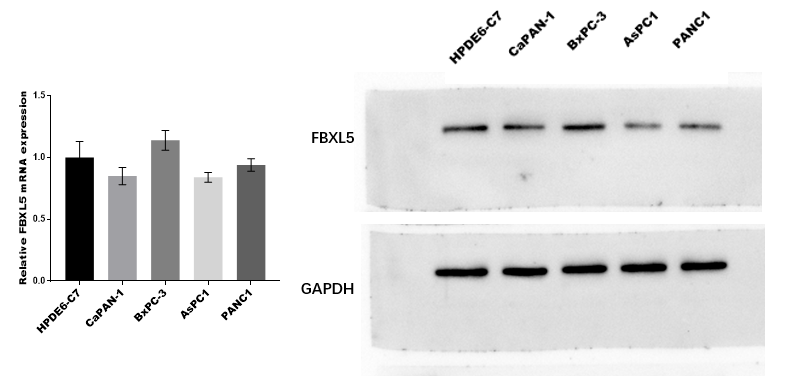

Supplement: Supplementary file 2 [file Data_Sheet_1.docx]
