# Supplementary material for: Downregulated F-Box/LRR-Repeat Protein 7 Facilitates Pancreatic Cancer Metastasis by Regulating Snail1 for Proteasomal Degradation
Source: Front Genet. 2021 Jun 24;12:650090. doi: 10.3389/fgene.2021.650090 (PMC8264591; doi:10.3389/fgene.2021.650090)

**Fig 2**

**fbxl7**


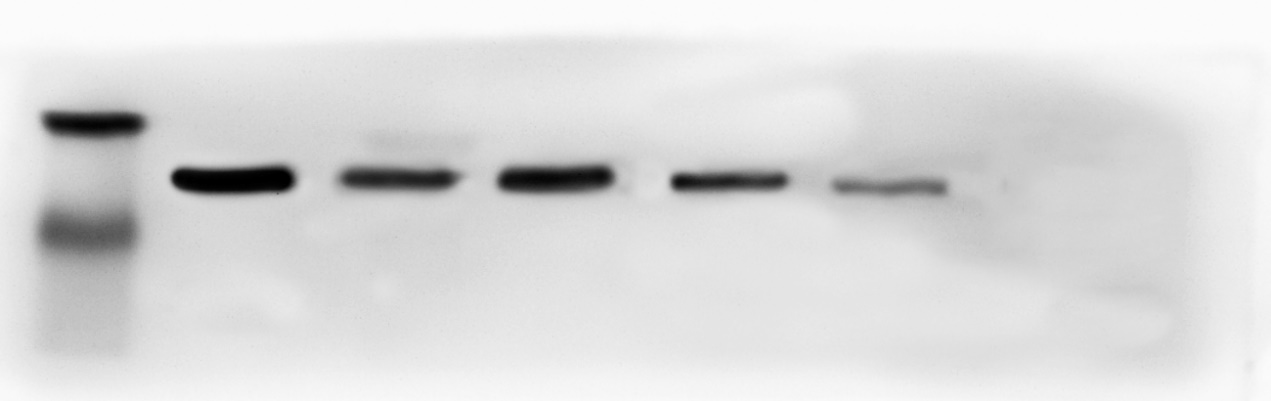


**fbxl7-KO**


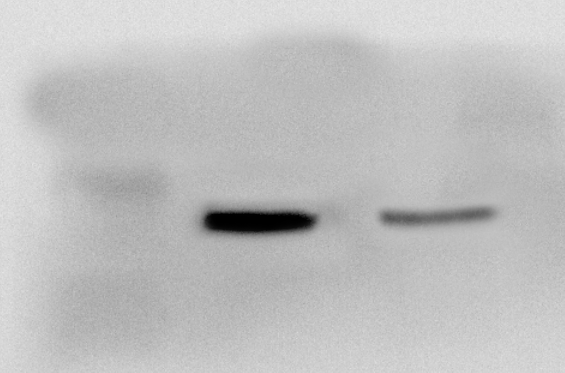


**fbxl7-ov**


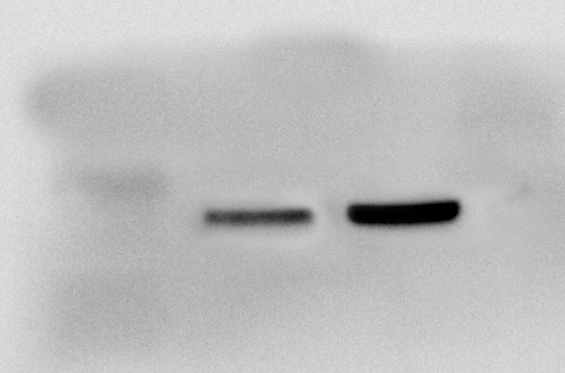


**GAPDH**


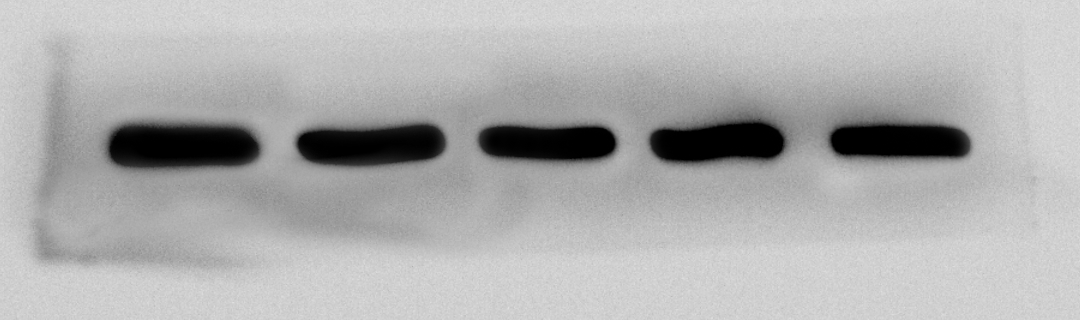


**GAPDH-KO-OVER**


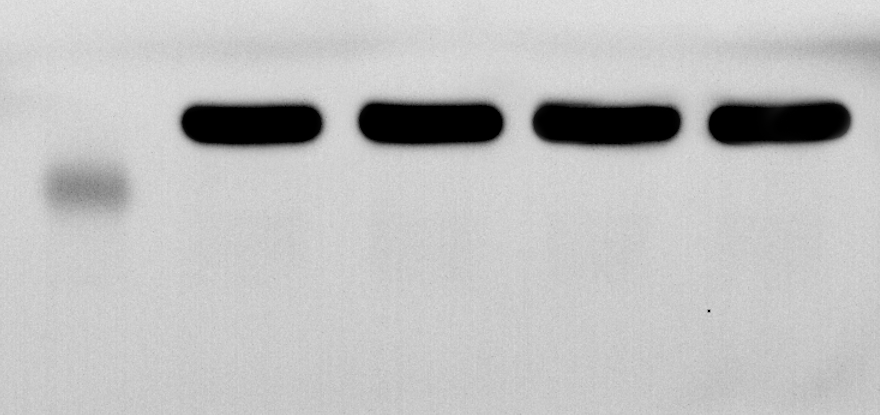


**Fig 3A**

**E-CADHERIN**


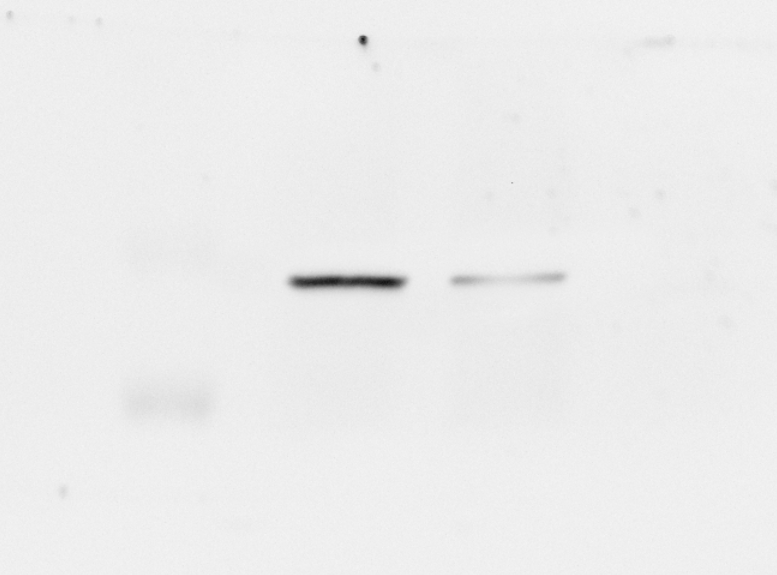


**GAPDH**


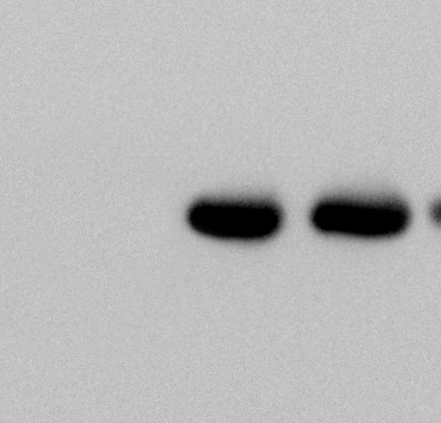


**N-CADHERIN**


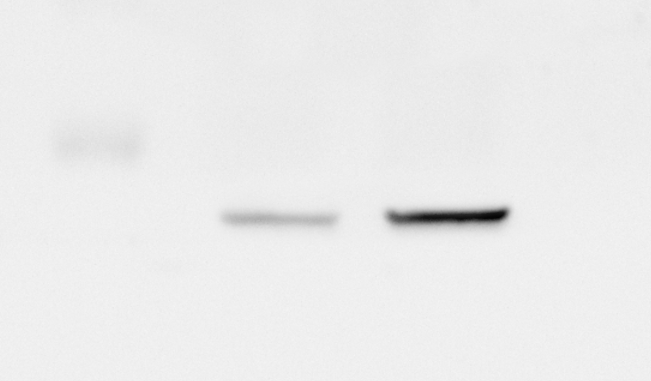


**snail1**


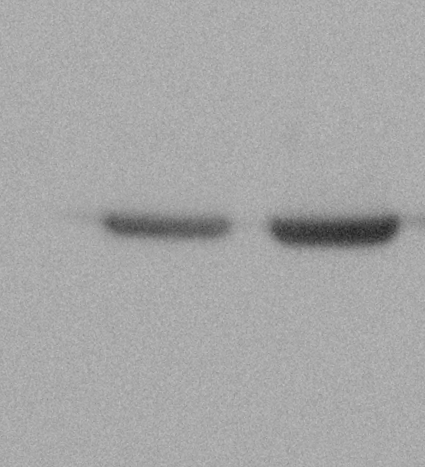


**snail2**


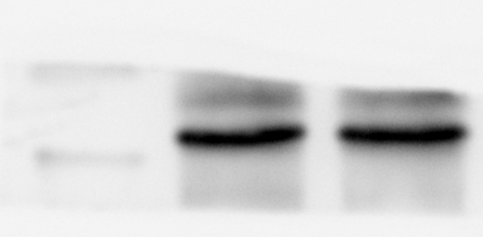


**Twist**


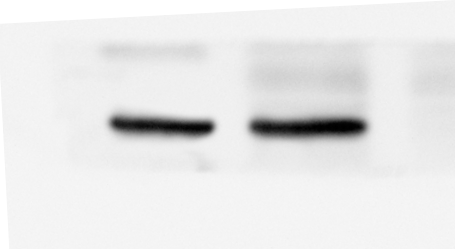


**VIMENTIN**


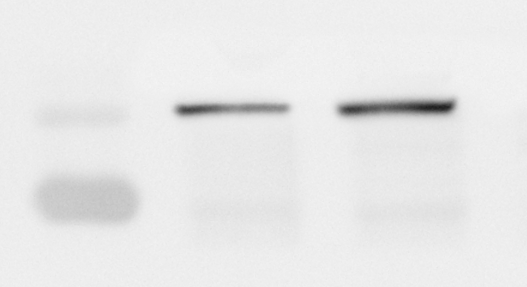


**zeb1**


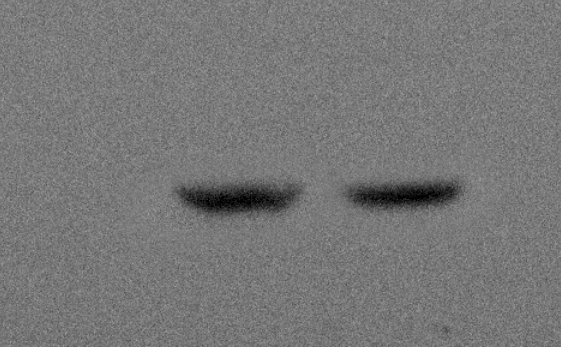


**Fig 3E**

**E-CADHERIN**


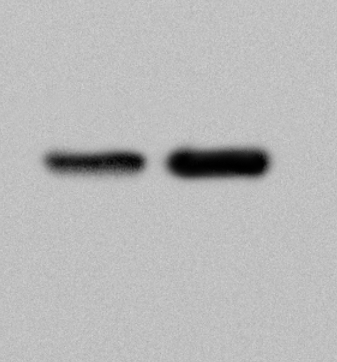


**GAPDH**


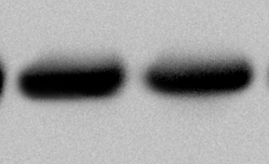


**N-CADHERIN**


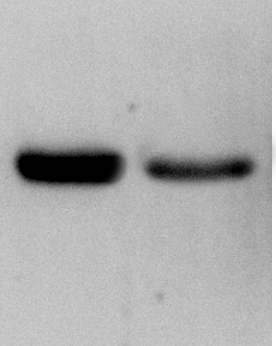


**SNAIL1**


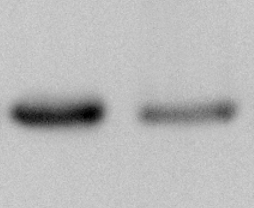


**SNAIL2**


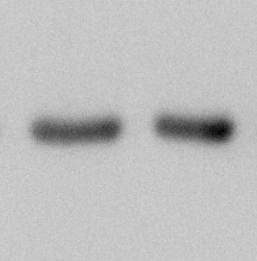


**TWIST**


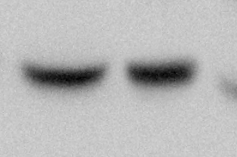


**VIMENTIN**


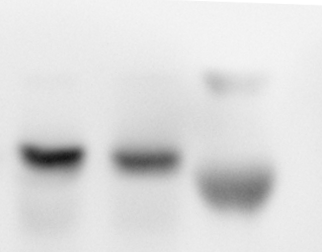


**ZEB1**


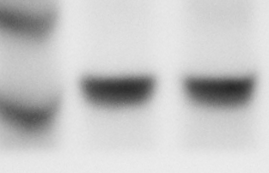


**Fig4**

**CHX**


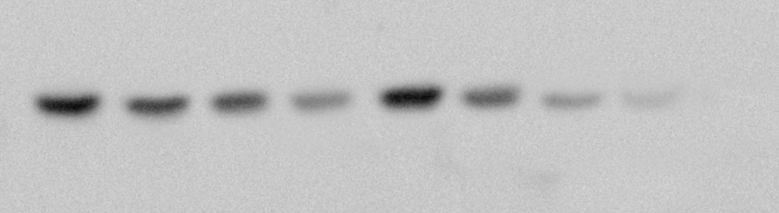


**CHX-GAPDH**


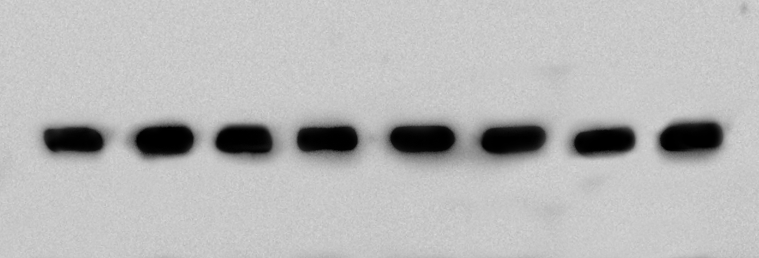


**FBXL7 OV +MG132**


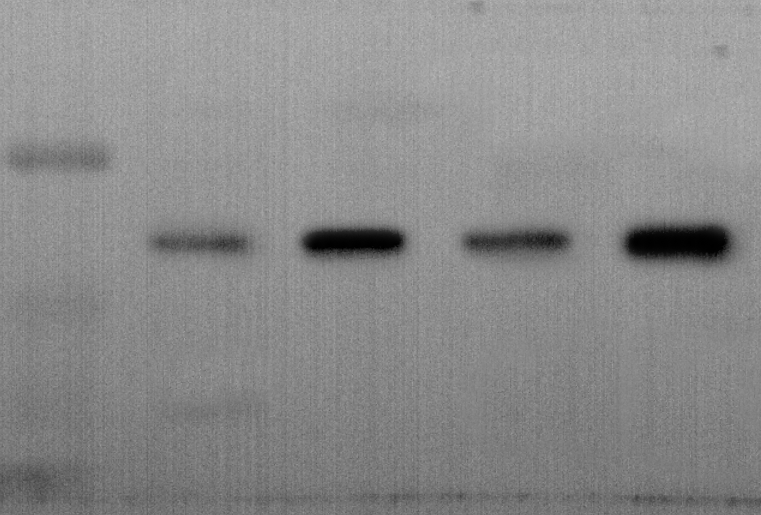


**FBXL7 OV +MG132-GAPDH**


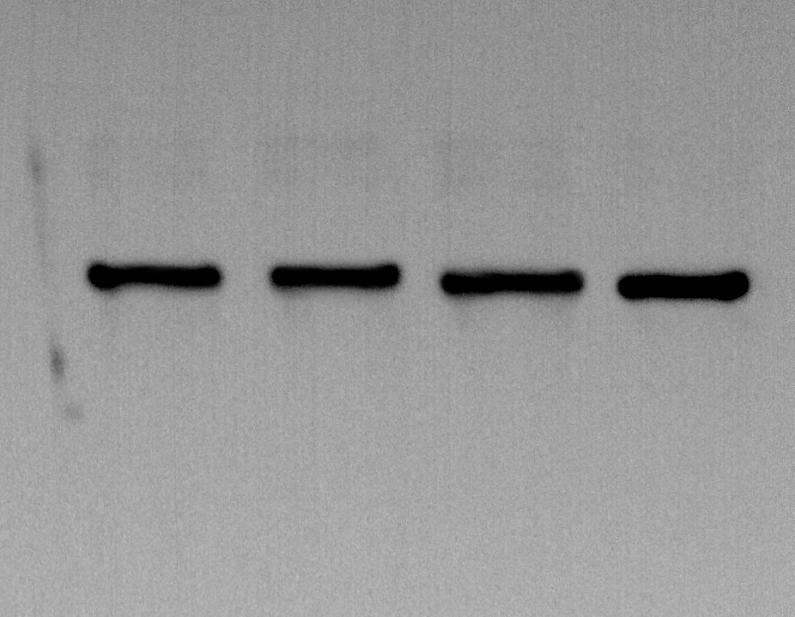


**FBXL7 OV +MG132-SNAIL1**


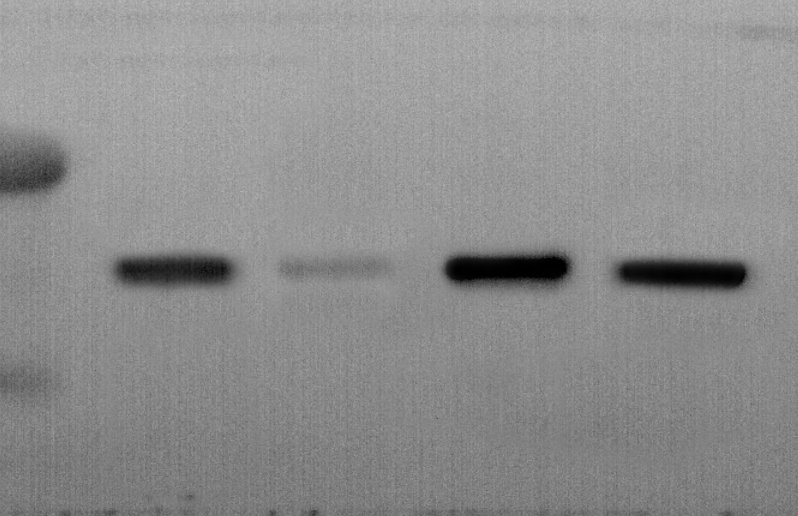


**IP FBXL7 IB FBXL7**


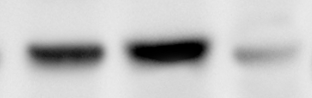


**IP FBXL7 IB SNAIL1**


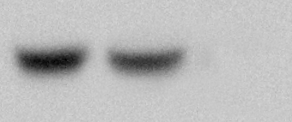


**IP Snail1 IB FBXL7**


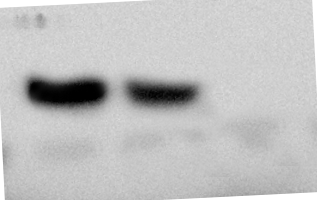


**IP Snail1 IB SNAIL1**


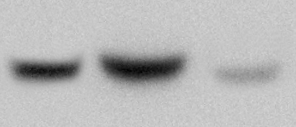

Supplement: Supplementary file 3 [file Data_Sheet_2.zip › WB/Western Blot raw data.docx]
