# Supplementary figures and images for: Downregulated F-Box/LRR-Repeat Protein 7 Facilitates Pancreatic Cancer Metastasis by Regulating Snail1 for Proteasomal Degradation
Source: Front Genet. 2021 Jun 24;12:650090. doi: 10.3389/fgene.2021.650090 (PMC8264591; doi:10.3389/fgene.2021.650090)

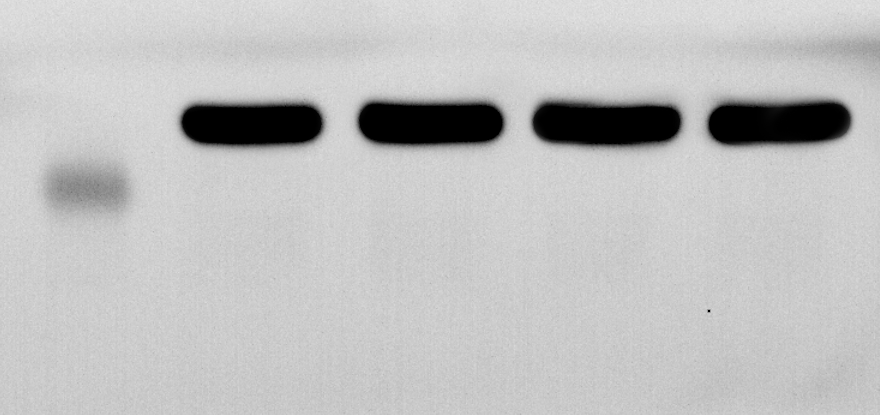

Supplement: Supplementary file 3 [file Data_Sheet_2.zip › WB/fig2/GAPDH-KO-OVER.tif]

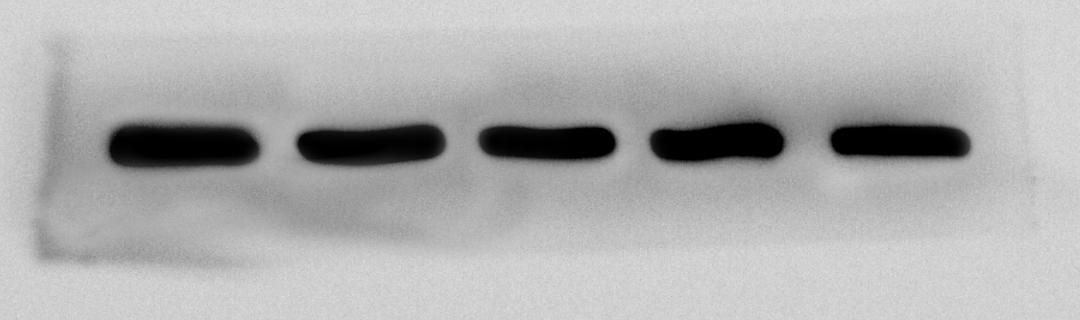

Supplement: Supplementary file 3 [file Data_Sheet_2.zip › WB/fig2/GAPDH.tif]

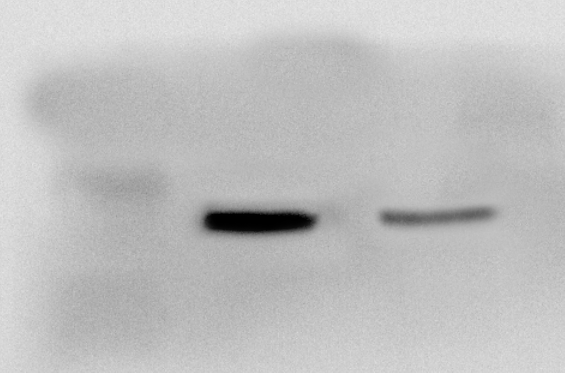

Supplement: Supplementary file 3 [file Data_Sheet_2.zip › WB/fig2/fbxl7-KO.tif]

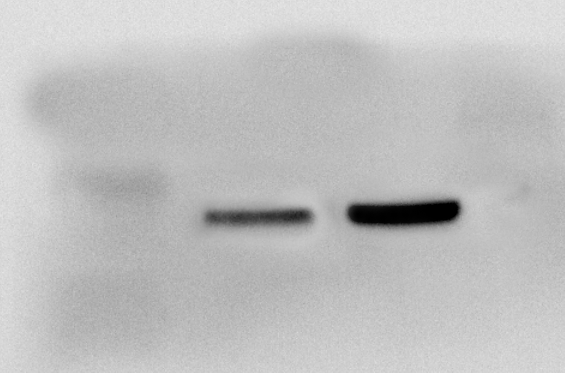

Supplement: Supplementary file 3 [file Data_Sheet_2.zip › WB/fig2/fbxl7-ov.tif]

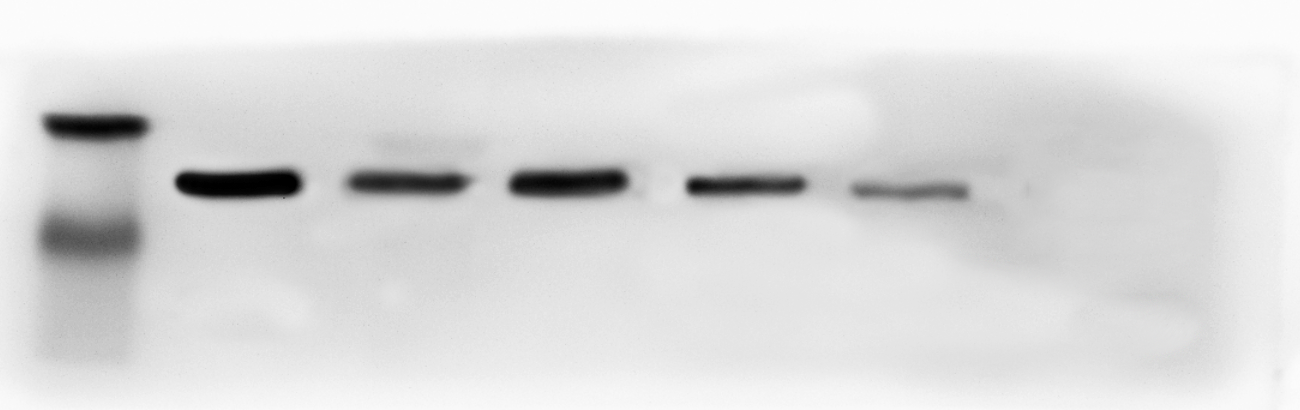

Supplement: Supplementary file 3 [file Data_Sheet_2.zip › WB/fig2/fbxl7.tif]

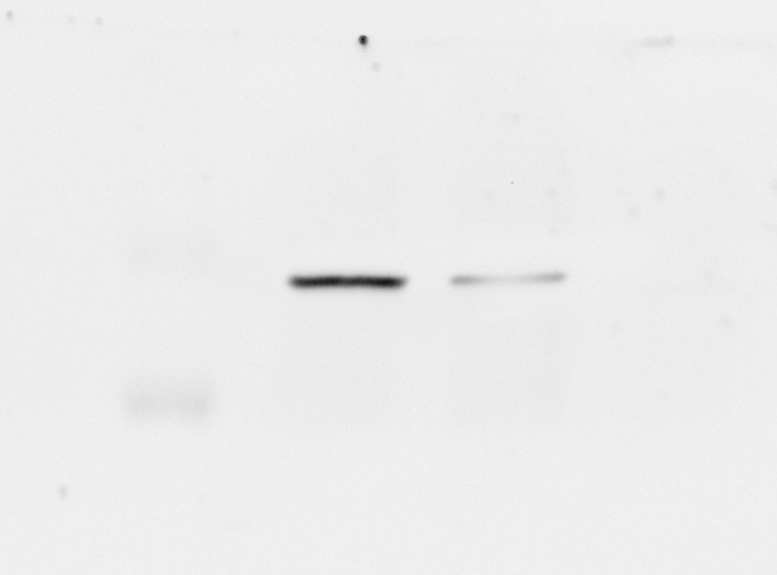

Supplement: Supplementary file 3 [file Data_Sheet_2.zip › WB/fig3/3A/E-CADHERIN.tif]

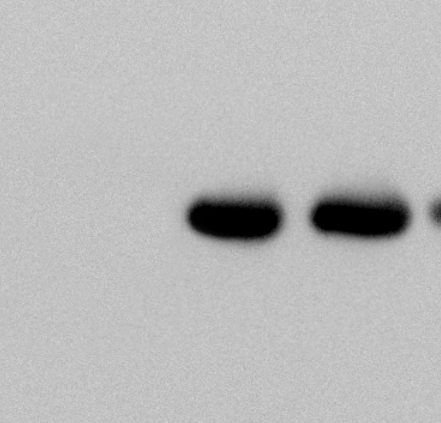

Supplement: Supplementary file 3 [file Data_Sheet_2.zip › WB/fig3/3A/GAPDH.tif]

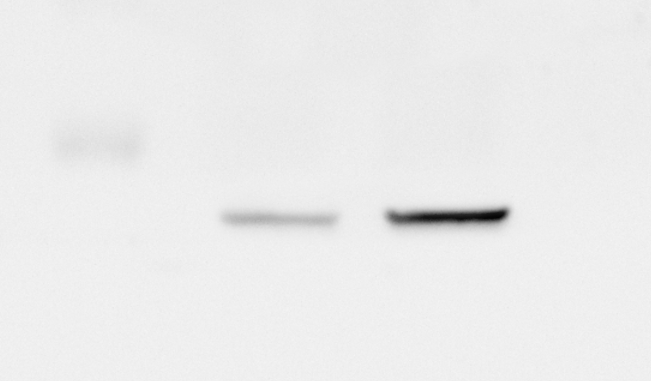

Supplement: Supplementary file 3 [file Data_Sheet_2.zip › WB/fig3/3A/N-CADHERIN.tif]

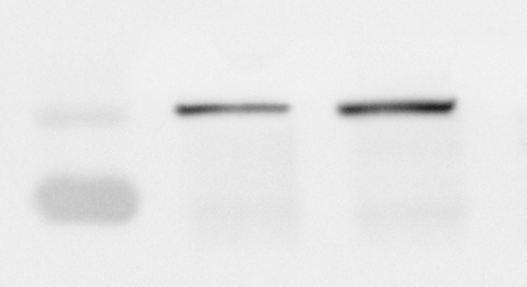

Supplement: Supplementary file 3 [file Data_Sheet_2.zip › WB/fig3/3A/VIMENTIN.tif]

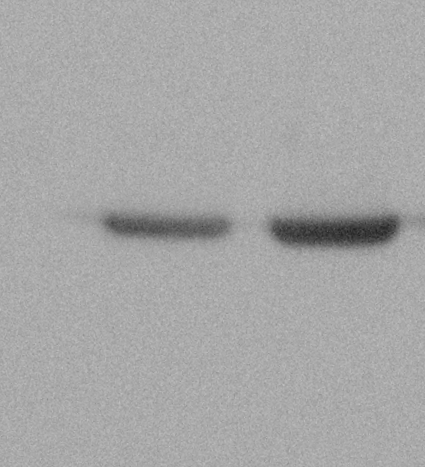

Supplement: Supplementary file 3 [file Data_Sheet_2.zip › WB/fig3/3A/snail1.tif]

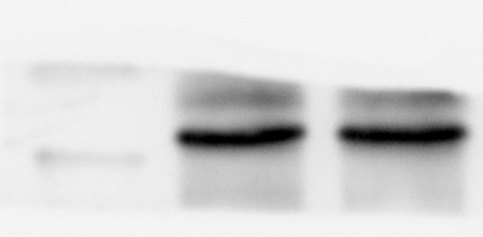

Supplement: Supplementary file 3 [file Data_Sheet_2.zip › WB/fig3/3A/snail2.tif]

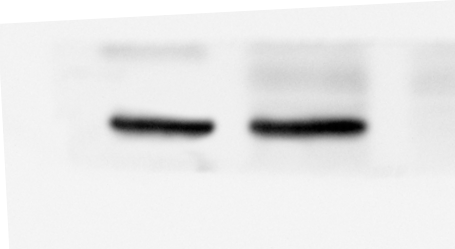

Supplement: Supplementary file 3 [file Data_Sheet_2.zip › WB/fig3/3A/twist.tif]

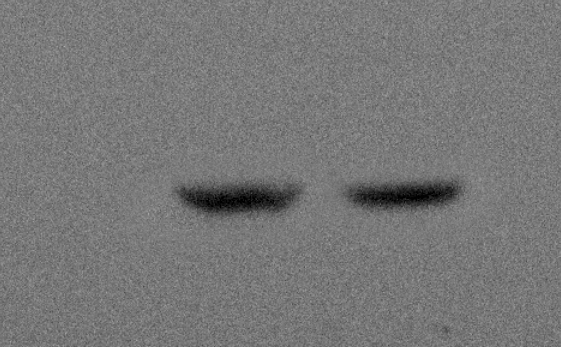

Supplement: Supplementary file 3 [file Data_Sheet_2.zip › WB/fig3/3A/zeb1.tif]

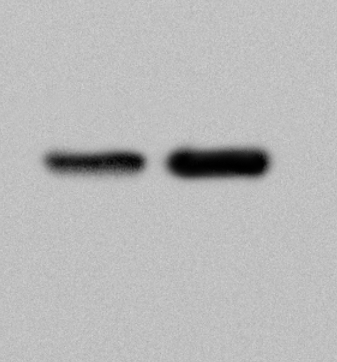

Supplement: Supplementary file 3 [file Data_Sheet_2.zip › WB/fig3/3E/E-CADHERIN.tif]

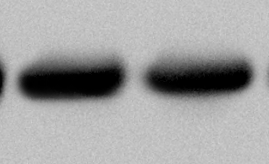

Supplement: Supplementary file 3 [file Data_Sheet_2.zip › WB/fig3/3E/GAPDH.tif]

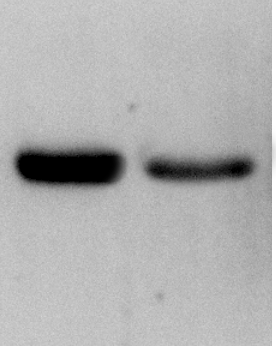

Supplement: Supplementary file 3 [file Data_Sheet_2.zip › WB/fig3/3E/N-CADHERIN.tif]

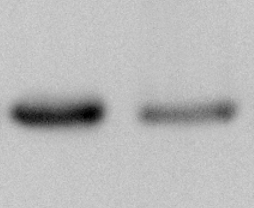

Supplement: Supplementary file 3 [file Data_Sheet_2.zip › WB/fig3/3E/SNAIL1.tif]

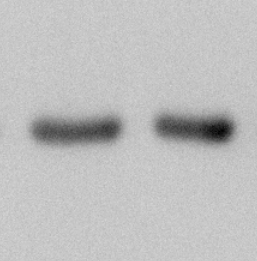

Supplement: Supplementary file 3 [file Data_Sheet_2.zip › WB/fig3/3E/SNAIL2.tif]

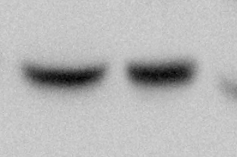

Supplement: Supplementary file 3 [file Data_Sheet_2.zip › WB/fig3/3E/TWIST.tif]

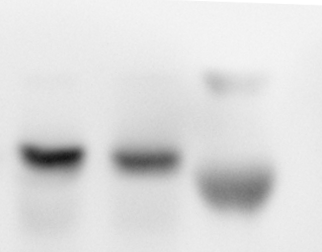

Supplement: Supplementary file 3 [file Data_Sheet_2.zip › WB/fig3/3E/VIMENTIN.tif]

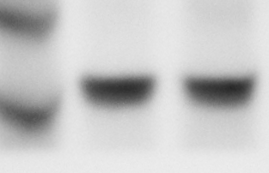

Supplement: Supplementary file 3 [file Data_Sheet_2.zip › WB/fig3/3E/ZEB1.tif]

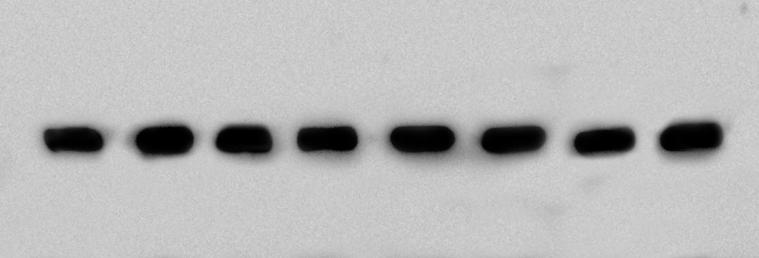

Supplement: Supplementary file 3 [file Data_Sheet_2.zip › WB/fig4/CHX-GAPDH.tif]

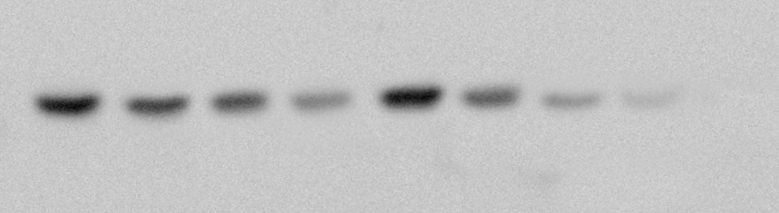

Supplement: Supplementary file 3 [file Data_Sheet_2.zip › WB/fig4/CHX.tif]

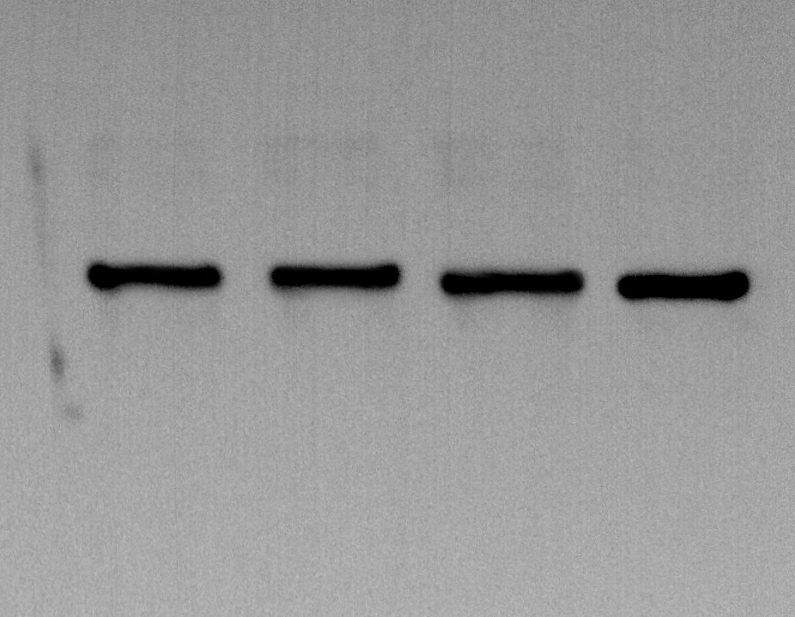

Supplement: Supplementary file 3 [file Data_Sheet_2.zip › WB/fig4/FBXL7 OV +MG132-GAPDH.tif]

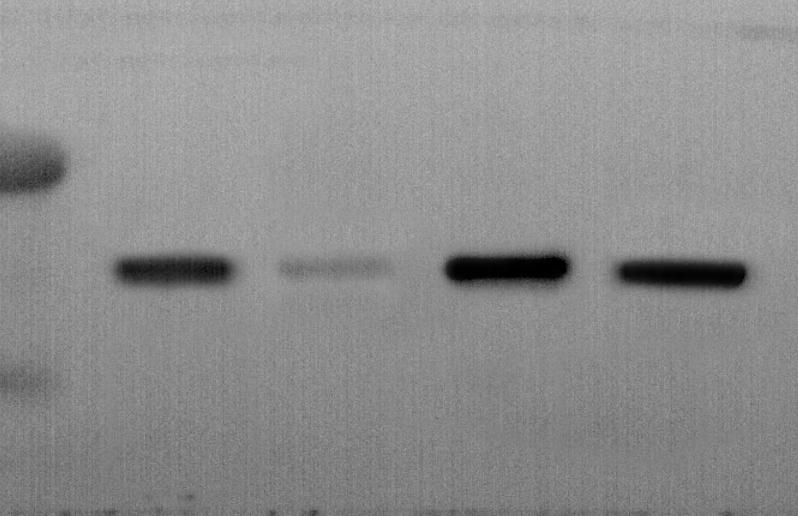

Supplement: Supplementary file 3 [file Data_Sheet_2.zip › WB/fig4/FBXL7 OV +MG132-SNAIL1.tif]

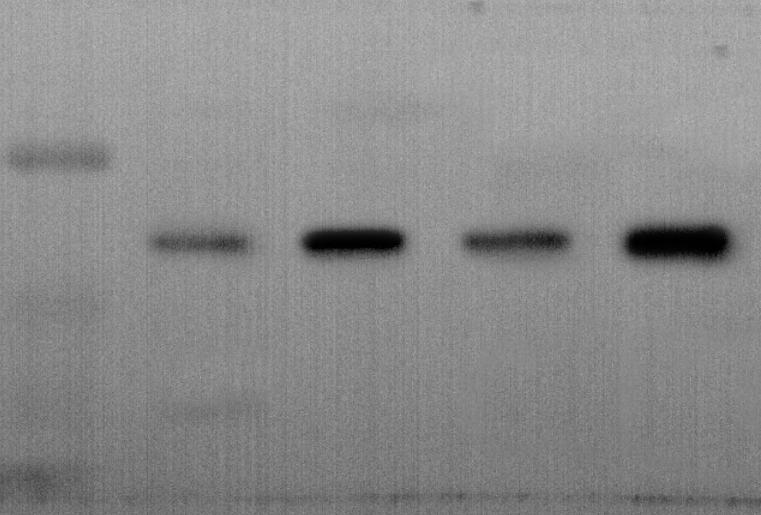

Supplement: Supplementary file 3 [file Data_Sheet_2.zip › WB/fig4/FBXL7 OV +MG132.tif]

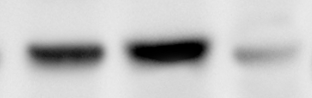

Supplement: Supplementary file 3 [file Data_Sheet_2.zip › WB/fig4/IP FBXL7 IB FBXL7.tif]

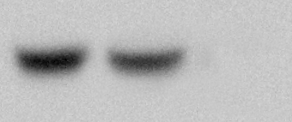

Supplement: Supplementary file 3 [file Data_Sheet_2.zip › WB/fig4/IP FBXL7 IB SNAIL1.tif]

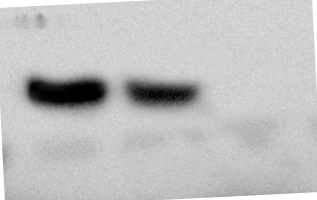

Supplement: Supplementary file 3 [file Data_Sheet_2.zip › WB/fig4/IP Snail1 IB FBXL7.tif]

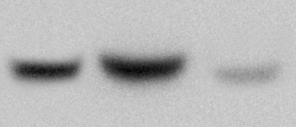

Supplement: Supplementary file 3 [file Data_Sheet_2.zip › WB/fig4/IP Snail1 IB SNAIL1.tif]
